# Supplementary material for: Comparative Analysis of Reconstitution and Solubility of Two Poly-L-Lactic Acid Fillers for Medical Applications
Source: Polymers (Basel). 2025 Jun 27;17(13):1778. doi: 10.3390/polym17131778 (PMC12251569; doi:10.3390/polym17131778)
Supplement: Supplementary file 1 [file polymers-17-01778-s001.zip › polymers-3660665-supplementary.pdf]

Supporting Information

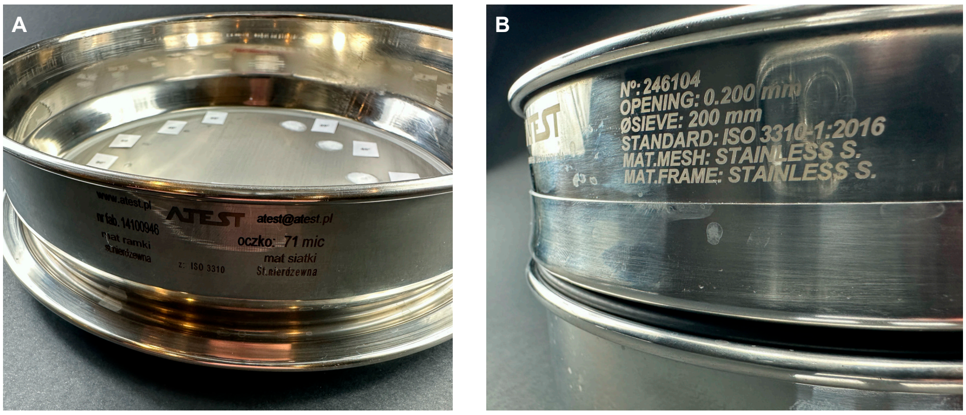

Figure S1. Images of the two standardized and certified micrometric sieves (ATEST, Poland) with pore sizes of 71 μm (A) and 200 μm (B).

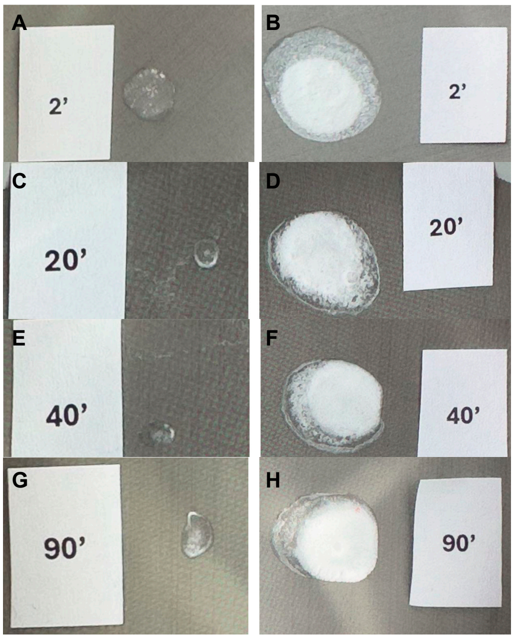

Figure S2. Sediment trace observed on the 71 μm sieve with the PLLA-LaSynPro™ and PLLA-SCA (8mL) solutions at different time points. PLLA-LaSynPro™: A, C, E, G slides PLLA-SCA: B, D, F , and H slides.

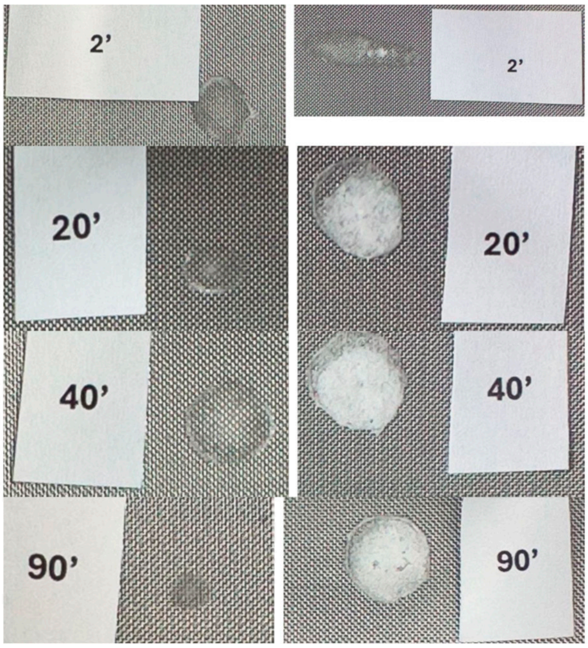

Figure S3. Sediment trace observed on the 200 μm sieve with the PLLA-LaSynPro™ and PLLA-SCA (8mL) solutions at different time points. PLLA-LaSynPro™: A, C, E, G slides PLLA-SCA: B, D, F , and H slides.

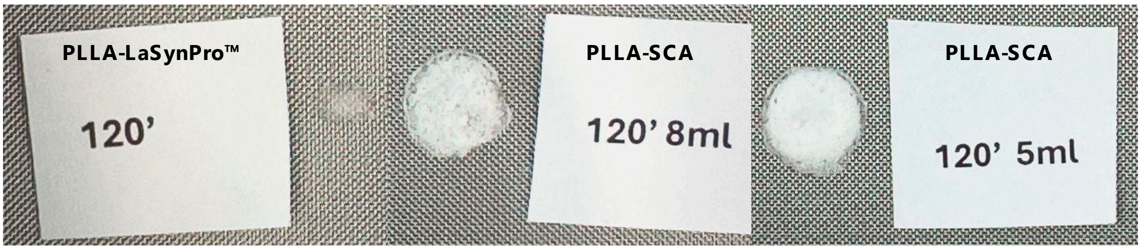

Figure S4. A comparison of the Sediment trace observed on the 200 μm sieve with the PLLA-LaSynPro™; PLLA-SCA (8mL); and PLLA-SCA (5mL) solutions at minute 120. Compared to the 8 mL PLLA-SCA solution, the 5 mL solution appeared slightly larger and thicker.
